# Bone marrow lesion and 5-year incident joint surgery in patients with knee osteoarthritis: a retrospective cohort study

**DOI:** 10.1186/s13018-024-04705-z

**Published:** 2024-05-21

**Authors:** Liang Lin, Jinshan Zhang, Hongyi Zhu, Zefeng Wang, Xiaofeng Liu, Yongquan Xu, Yangzhen Fang, Zhenyu Lin, Yongqiang Zheng

**Affiliations:** 1 Department of Orthopedics, Jinjiang Municipal Hospital, Fujian, China; 2 Department of Orthopedics, Shanghai Jiao Tong University Affiliated Sixth People’s Hospital, Shanghai, China; 3 Institute of Clinical Research, National Center for Orthopaedics, Shanghai Sixth People’s Hospital, Shanghai, China; 4 Clinical Research Center for Orthopaedic Trauma and Reconstruction of Fujian Province, Jinjiang Municipal Hospital, Fujian, China

**Keywords:** Knee osteoarthritis, Bone marrow lesion, Joint surgery, Association, Predictive value

## Abstract

**Background:**

It is beneficial for society to discover the risk factors associated with surgery and to carry out some early interventions for patients with these risk factors. Few studies specifically explored the relationship between bone marrow lesions (BMLs) and long-term incident joint surgery.

**Objective:**

To investigate the association between BML severity observed in knee osteoarthritis (OA) patients’ first MRI examination and incident knee surgery within 5 years. Additionally, to assess the predictive value of BMLs for the incident knee surgery.

**Design:**

Retrospective cohort study.

**Methods:**

We identified patients diagnosed with knee OA and treated at our institution between January 2015 and January 2018, and retrieved their baseline clinical data and first MRI examination films from the information system. Next, we proceeded to determine the Max BML grades, BML burden grades and Presence BML grades for the medial, lateral, patellofemoral, and total compartments, respectively. Multi-variable logistic regression models examined the association of the BML grades with 5-year incident knee surgery. Positive and negative predictive values (PPVs and NPVs) were determined for BML grades referring to 5-year incident knee surgery.

**Results:**

Totally, 1011 participants (knees) were found eligible to form the study population. Within the 5 years, surgery was performed on 74 knees. Max BML grade 2 and grade 3 of medial, patellofemoral and total compartments were strongly and significantly associated with incident surgery. None of the BML grades from lateral compartment was associated with incident surgery. The PPV was low and NPV was high for BMLs.

**Conclusions:**

BMLs found in the first MRI examination were associated with 5-year incident joint surgery, except for those allocated in lateral compartments. The high NPVs imply that patients without BMLs have a low risk of requiring surgery within 5 years.

**Supplementary Information:**

The online version contains supplementary material available at 10.1186/s13018-024-04705-z.

## Introduction

Osteoarthritis (OA) is a prevalent joint disorder characterized by the progressive degradation of cartilage, leading to pain, stiffness, and functional limitations [1, 2] Although cartilage damage has long been recognized as a hallmark of OA, emerging research has shed light on the role of bone marrow lesions (BMLs) in this condition [3, 4].

BMLs are abnormal changes observed within the bone marrow, often adjacent to areas of cartilage degeneration, and are typically identified through magnetic resonance imaging (MRI) scans, where they appear as areas of increased signal intensity within the bone marrow [5]. Even though their exact etiology remains unclear, emerging evidence suggests that BMLs result from a complex interplay between mechanical factors, inflammation, and microtrauma within the joint [3, 6]. These factors contribute to the disruption of the delicate balance between bone formation and resorption, leading to focal areas of abnormal bone remodeling.

Clinically, the presence of BMLs in OA has been associated with joint pain [7]. However, there is insufficient strong evidence to support the idea that the exacerbation of pain caused by BMLs would further drive OA patients to seek medical care, particularly for joint surgery. The large amount of medical expenses generated by surgery will cause a greater burden on the entire medical system [1, 8]. Therefore, it would be beneficial for society to discover the risk factors associated with surgery and to carry out some early interventions for patients with these risk factors. A few previous studies specifically explored the relationship between BMLs, and long-term surgery rates indicated that knee OA patients with BMLs had a higher risk of knee replacement surgery^9^–^11^>. Nevertheless, they were published ten years ago when BMLs were measured using 1.5T equipment, and BML was not clearly defined, highlighting the necessity for updating the classification criteria. Moreover, these studies did not take patellofemoral joint into account and the sample sizes were small, making it difficult to draw solid conclusions.

In the current study, we aimed to investigate the association between BML severity observed in patient first MRI examination and incident knee surgery within 5 years through a retrospective analysis on a large group of knee OA patients. Furthermore, to assess the predictive value of BMLs for incident knee surgery over a 5-year timeframe.

## Patients and methods

### Study population

We identified patients diagnosed with knee OA and treated at our institution (a regional tertiary hospital) between January 1st 2015 and January 1st 2018 using the hospital information system. Written informed consent was obtained from all patients once they have administered in our hospital that their medical data may be used for scientific analysis but will be kept anonymous. Patient data were collected on May 2nd 2023; authors did not have access to information that could identify individual participants during or after data collection.

The current study was done on knee level. To be eligible for inclusion, knees had to meet the following criteria: they belonged to patients aged over 40 years, were reported to have knee pain, were confirmed to have radiographic knee OA (Kellgren & Lawrence (KL) grade > = 2) through radiographs and had magnetic resonance imaging (MRI) films available. The year when the patient underwent their initial MRI examinations following the diagnosis of knee OA was defined as the baseline for this study. Knees were excluded if they had injuries resulting in structural damage after the baseline assessment, had other knee joint conditions or forms of arthritis, lacked radiographs or MRI exams, or if the patient had scheduled knee surgery at the baseline evaluation. In cases where a patient had bilateral knee OA, we selected the knee that was first diagnosed with OA. If both knees were diagnosed at the same clinical visit, we randomly selected one for inclusion in the study.

### Baseline patient data

After a patient was included in the study, their baseline clinical data and imaging films were retrieved from the information system. The baseline clinical data consisted of demographic information (age, sex, BMI (body mass index), education, affected side of knee OA and OA in other joints) and pain level of the past week was assessed using the numeric rating scale (NRS) score (0–10, 0 indicates no pain; patients were asked when receiving MRI examinations). Two researchers (J.Z. and Z.W.), who were unaware of the patients’ clinical information, independently reviewed weight-bearing knee radiographs taken at baseline (or before) and assigned scores using the KL classification system [9]. In cases where there were discrepancies, the two researchers held a consensus meeting to resolve differences and reach agreements.

### MRI scan and BML

All the knees were imaged using a 3.0T MRI unit in the sagittal plane (resolution 1.5; slice thickness 2 mm) in our hospital. Two researchers (J. Z. and Z.W.), who were blinded to the patients’ clinical information, independently reviewed the baseline T1 and T2 MRI films. They assigned BML grades according to the standardized MRI Osteoarthritis Knee Score (MOAKS) system [10].

In the MOAKS system, a BML is defined as an ill-defined trabecular bone signal that appears hypointense on T1-weighted imaging and hyperintense on T2-weighted fat-saturated imaging. The researchers assessed BMLs in each subregion, which consisted of six subregions for the femur, two for the tibia and two for patella, as specified by the MOAKS system. BMLs, including cysts, were graded on a scale of 0 to 3 based on the percentage of subregional volume involved: grade 0 indicated no BML, grade 1 indicated involvement of less than 33% of the subregional volume, grade 2 indicated involvement of 33–66% of the subregional volume, and grade 3 indicated involvement of more than 66% of the subregional volume. If multiple BMLs were present in a single subregion, their volumes were combined into a single percentage. We proceeded to determine the Max BML grade for the medial, lateral, patellofemoral, and total compartments, respectively. This was determined by selecting the highest grade among all corresponding subregions. Building upon this, we created BML burden grades by summing the Max BML grades of the medial, lateral and patellofemoral compartments. As a result, the BML burden grade ranged from 0 to 9. However, among included knees, there was no knee graded 8 and 9, and prevalence of grade 5 (2 knees), 6 (1 knee) and 7(1 knee) was very low. Therefore, we incorporated grade 5–7 into grade 4 group. Additionally, we assigned Presence BML grades for each compartment: an Presence BML grade of 0 indicated the absence of BML, while a grade of 1 indicated the presence of BML.

### Outcome measure

The primary outcome is incident knee surgery within 5 years from baseline. At the 5-year mark, we reached out to all patients and inquired whether they had undergone any knee surgery specifically for OA in the knee that had been subject to the baseline MRI. The knee surgery included total/unicompartment knee replacement, arthroscopic procedures and high tibial osteotomy. The exact surgery indication for each individual could not be reached. In our hospital, knee surgery was indicated for OA patients when conservative treatments, such as medication, physical therapy, and lifestyle modifications, have failed to provide adequate relief from symptoms, and the patient’s quality of life is significantly impacted by knee pain and dysfunction. The first surgery was used if multiple surgeries were done on one knee. To verify this information, we cross-referenced patient medical care records in our hospital system.

We used radiographic progression indicators as the secondary outcomes to further support the role of BMLs in disease development. We identified patients who had received radiographic exams at 4 to 6 years from baseline and created radiographic progression indicators according to KL classification system, i.e., KL progression (KL progressed 1 grade or more vs. no change). Knees with baseline KL grade 4 were excluded.

### Statistical analysis

We tabulated descriptive statistics to summarize the characteristics of the subjects. We built logistic regression models to assess the relationship between baseline Max BML/ BML burden/Presence BML grades and the incident surgery over the 5-year period for medial, lateral, patellofemoral and total compartments, respectively. Crude odds ratios (ORs) and adjusted ORs (adjusted for baseline age, sex, BMI and KL grade) were calculated, as well as their 95%CIs. The reference group for comparison was baseline Max BML/BML burden/Presence BML grade 0. The co-variates were chosen according to previous literature that those could be confounding factors [11]. We used similar logistic regression models to assess the relationship between baseline Max BML/ BML burden/Presence BML grades and KL progression.

Next, we determined the positive and negative predictive values (PPV and NPV) of baseline BML grades for predicting the incidence of surgery over the 5-year period. To do this, we applied multiple cutoffs on the Max BML grades to obtain dichotomized categories, i.e., Max BML grade 1 or higher, Max BML grade 2 or higher and Max BML grade 3. We did this for all the compartments separately and did the same for the BML burden grades.

Knees with baseline characteristic data missing were imputed by the data from prior clinical visits (e.g., age, sex and BMI). While 78 knees had missing values in baseline NRS score were excluded from the descriptive analysis. There was no missing value in the primary outcome measure (surgery information) as we reached out to patients, and most were also available in the healthcare information system. All statistical analyses were performed using the SPSS software (IBM, Chicago, USA). Statistical significance was defined as *P* < 0.05.

## Results

**Fig. 1:**
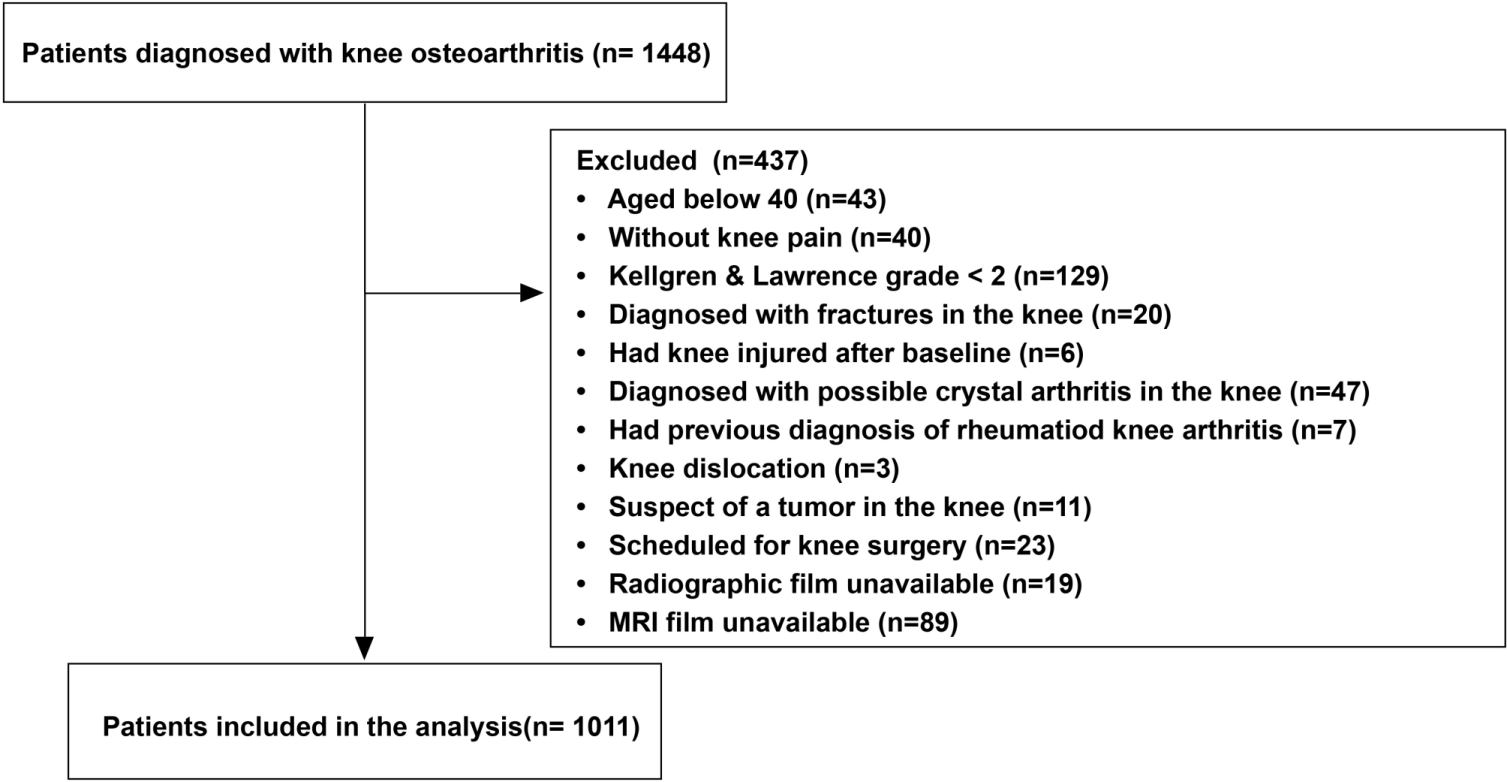
Patient inclusion procedure

In total, 1448 patients were diagnosed with knee OA and identified; of those 437 were excluded, the most common reason for exclusion was KL grade < 2 at baseline (*n* = 129). See detailed patient inclusion procedure in Fig. 1. As a result, 1011 participants (knees) were found eligible to form the study population with a mean (SD) age of 61 (8.7), 73% female and a mean (SD) BMI of 25.1 (3.5). Within the 5 years, surgery was performed by 6 surgeons on 74 knees; specifically, 25 knees had total knee replacement, 8 had uni-compartment replacement, 32 had arthroscopic procedures (for removing damaged cartilage, repairing degenerative ligament, meniscus tear, partial meniscectomy and debridement) and 9 had high tibial osteotomy. The baseline characteristics of the knees with/without surgery are presented in Table 1.

**Table 1:** Baseline demographic and radiographic characteristics of the knees received/did not receive surgery within the 5 years

Characteristics	Knee surgery (*n* = 74)	No knee surgery (*n* = 937)
Age, years, mean (SD)	61.3 (8.0)	61.2 (8.7)
Sex, female (%)	56 (76)	685 (73)
BMI, kg/m^2^, mean (SD)	24.5 (2.8)	25.2 (3.6)
Bilateral OA, yes (%)	56 (76)	770 (82)
Education, high school or higher (%)	22 (30)	377 (40)
Smoking, n (%)	14 (19)	163 (17)
Have hand OA, n (%)	12 (16)	56 (6)
Have hip OA, n (%)	7 (10)	96 (10)
Time from OA diagnosis to enrollment, months, mean (SD)	54.5 (25.7)	51.8 (27.7)
NRS pain score, 0–10, mean (SD)※	1.7 (1.3)	1.7 (1.2)
KL grade, n (%)		
2	54 (73)	657 (70)
3	18 (24)	274 (29)
4	2 (3)	6 (1)

SD, standard deviation; BMI, body mass index; OA, osteoarthritis; NRS, numeric rating scale; KL, Kellgren & Lawrence

※78 knees had missing values in NRS score

### The association between BML and 5-year incident surgery

For medial compartment, Max BML grade 2 and grade 3 were strongly and significantly associated with incident surgery with crude ORs (95%CI) of 3.6 (1.5 to 8.7) and 4.2 (1.1 to 15.8), respectively. The adjusted ORs (95%CI) were similar, which were 3.8 (1.6 to 9.2) and 4.6 (1.2 to 17.4) for Max BML grade 2 and grade 3, respectively. Presence BML grade 1 was marginally associated with incident surgery when adjusted for age, sex, BMI and KL grade (Table 2).

None of the BML grades from lateral compartment was associated with incident surgery (Table 2).

**Table 2:** Association between baseline BML status and incident surgery within 5 years

	Crude OR (95%CI)	P values	¶Adjusted OR (95%CI)	P values
**Medial compartment**				
Max BML grade 0, *n* = 660	Reference		Reference	
Max BML grade 1, *n* = 300	1.3 (0.7 to 2.2)	0.353	1.3 (0.8 to 2.2)	0.344
Max BML grade 2, *n* = 37	3.6 (1.5 to 8.7)	**0.004**	3.8 (1.6 to 9.2)	**0.003**
Max BML grade 3, *n* = 14	4.2 (1.1 to 15.8)	**0.032**	4.6 (1.2 to 17.4)	**0.026**
Presence BML grade 0, *n* = 660	Reference		Reference	
Presence BML grade 1, *n* = 351	1.6 (0.9 to 2.6)	0.052	1.6 (1.0 to 2.6)	**0.049**
**Lateral compartment**				
Max BML grade 0, *n* = 821	Reference		Reference	
Max BML grade 1, *n* = 154	1.1 (0.6 to 2.0)	0.833	1.1 (0.6 to 2.1)	0.789
Max BML grade 2, *n* = 31	0.4 (0.1 to 3.1)	0.401	0.4 (0.1 to 3.2)	0.407
Max BML grade 3, *n* = 5	3.2 (0.3 to 28.8)	0.305	3.5 (0.4 to 32.7)	0.265
Presence BML grade 0, *n* = 821	Reference		Reference	
Presence BML grade 1, *n* = 190	1.0 (0.5 to 1.8)	0.977	1.0 (0.6 to 1.9)	0.934
**Patellofemoral compartment**				
Max BML grade 0, *n* = 410	Reference		Reference	
Max BML grade 1, *n* = 548	1.2 (0.7 to 2.1)	0.407	1.3 (0.7 to 2.2)	0.379
Max BML grade 2, *n* = 40	4.9 (2.1 to 11.5)	**< 0.001**	5.1 (2.1 to 12.0)	**< 0.001**
Max BML grade 3, *n* = 13	7.5 (2.1 to 26.1)	**0.002**	7.9 (2.2 to 27.9)	**0.001**
Presence BML grade 0, *n* = 410	Reference		Reference	
Presence BML grade 1, *n* = 601	1.6 (0.9 to 2.6)	0.087	1.6 (0.9 to 2.6)	0.078
**Total compartments**				
Max BML grade 0, *n* = 218	Reference		Reference	
Max BML grade 1, *n* = 657	2.1 (0.9 to 4.8)	0.072	2.1 (0.9 to 4.8)	0.068
Max BML grade 2, *n* = 106	5.8 (2.3 to 14.4)	**< 0.001**	6.0 (2.4 to 15.0)	**< 0.001**
Max BML grade 3, *n* = 30	9.2 (3.0 to 28.5)	**< 0.001**	9.9 (3.2 to 31.3)	**< 0.001**
Presence BML grade 0, *n* = 218	Reference		Reference	
Presence BML grade 1, *n* = 793	2.8 (1.3 to 6.1)	**0.012**	2.8 (1.3 to 6.2)	**0.010**
BML burden grade 0, *n* = 218	Reference		Reference	
BML burden grade 1, *n* = 430	1.9 (0.8 to 4.5)	0.127	1.9 (0.8 to 4.6)	0.123
BML burden grade 2, *n* = 247	3.4 (1.4 to 8.0)	**0.005**	3.5 (1.5 to 8.2)	**0.005**
BML burden grade 3, *n* = 81	5.2 (2.0 to 13.8)	**0.001**	5.4 (2.0 to 14.4)	**0.001**
BML burden grade 4, *n* = 35	3.9 (1.1 to 14.1)	**0.038**	4.2 (1.2 to 15.4)	**0.029**

BML, bone marrow lesion; CI, confidence interval

¶ Adjusted for age, sex, BMI and KL grade

For patellofemoral compartment, Max BML grade 2 and grade 3 were also strongly and significantly associated with incident surgery with crude ORs (95%CI) of 4.9 (2.1 to 11.5) and 7.5 (2.1 to 26.1), respectively. The adjusted ORs (95%CI) were similar, which were 5.1 (2.1 to 12.0) and 7.9 (2.2 to 27.9) for Max BML grade 2 and grade 3, respectively. The association between BML presence and incident surgery was weak and statistically insignificant (Table 2).

The results for Max BML grades of the total compartments were similar to those of patellofemoral compartment; while the association between Presence BML grade 1 and incident surgery became stronger and statistically significant, with crude and adjusted ORs (95%CI) of 2.8 (1.3 to 6.1) and 2.8 (1.3 to 6.2), respectively. Besides, steady and strong associations were found between BML burden grade 2, 3 and 4 and incident surgery, with estimated ORs ranged from 3.4 to 5.4 (Table 2).

### Predictive values of BML for 5-year incident surgery

Table 3 presents the PPV and NPV of each cutoff on the Max BML/BML burden grade from each compartment. In general, the PPV was low and NPV was high for all the BML grades. Among those, Max BML grade in patellofemoral compartment presented best predictive ability for 5-year incident surgery.

**Table 3:** Positive/negative predictive values of baseline BML status for incident surgery within 5 years

	No. of surgery/total	PPV	NPV
**Medial compartment**			
Max BML grade 1 or higher	33/351	9%	94%
Max BML grade 2 or higher	10/51	20%	93%
Max BML grade 3	3/14	21%	93%
**Lateral compartment**			
Max BML grade 1 or higher	14/190	7%	93%
Max BML grade 2 or higher	2/36	6%	93%
Max BML grade 3	1/5	20%	93%
**Patellofemoral compartment**			
Max BML grade 1 or higher	51/601	8%	94%
Max BML grade 2 or higher	13/53	24%	94%
Max BML grade 3	4/13	31%	93%
**Total compartments**			
Max BML grade 1 or higher	67/793	8%	97%
Max BML grade 2 or higher	24/136	18%	94%
Max BML grade 3	7/30	23%	93%
BML burden grade 1 or higher	67/793	8%	97%
BML burden grade 2 or higher	41/363	11%	95%
BML burden grade 3 or higher	16/116	14%	94%
BML burden grade 4	4/35	11%	93%

BML, bone marrow lesion; PPV, positive predictive value; NPV, negative predictive value

### The association between BML and KL progression

517 knees with radiographs available at 4 to 6 years were included in the analysis; among them 253 (25%) knees were identified with KL progression. Baseline characteristics between included and excluded knees are presented in Appendix table. The association between baseline BML status and KL progression was similar to the above (Table 4).

**Table 4:** Association between baseline BML status and KL progression

	Crude OR (95%CI)	P values	¶Adjusted OR (95%CI)	P values
**Medial compartment**				
Max BML grade 0, *n* = 347	Reference		Reference	
Max BML grade 1, *n* = 150	0.9 (0.7 to 1.5)	0.965	1.0 (0.7 to 1.4)	0.925
Max BML grade 2, *n* = 16	7.7 (1.7 to 34.5)	**0.007**	8.0 (1.8 to 36.3)	**0.007**
Max BML grade 3, *n* = 4	8.3 (1.3 to 32.1)	**0.002**	8.6 (1.5 to 34.2)	**0.006**
Presence BML grade 0, *n* = 347	Reference		Reference	
Presence BML grade 1, *n* = 170	1.2 (0.82 to 1.7)	0.368	1.2 (0.8 to 1.7)	0.379
**Lateral compartment**				
Max BML grade 0, *n* = 412	Reference		Reference	
Max BML grade 1, *n* = 86	1.2 (0.8 to 1.9)	0.428	1.2 (0.7 to 1.9)	0.431
Max BML grade 2, *n* = 17	0.6 (0.2 to 1.6)	0.281	0.6 (0.2 to 1.8)	0.640
Max BML grade 3, *n* = 2	Too small sample		Too small sample	
Presence BML grade 0, *n* = 264	Reference		Reference	
Presence BML grade 1, *n* = 253	1.0 (0.7 to 1.6)	0.893	1.0 (0.6 to 1.6)	0.841
**Patellofemoral compartment**				
Max BML grade 0, *n* = 202	Reference		Reference	
Max BML grade 1, *n* = 284	1.1 (0.9 to 2.0)	0.118	1.4 (0.9 to 2.1)	0.078
Max BML grade 2, *n* = 22	3.7 (1.7 to 7.9)	**0.001**	3.8 (1.8 to 8.3)	**< 0.001**
Max BML grade 3, *n* = 9	5.5 (2.0 to 15.4)	**0.001**	5.9 (2.1 to 16.6)	**0.001**
Presence BML grade 0, *n* = 202	Reference		Reference	
Presence BML grade 1, *n* = 315	1.1 (0.8 to 1.6)	0.487	1.2 (0.8 to 1.7)	0.413
**Total compartments**				
Max BML grade 0, *n* = 109	Reference		Reference	
Max BML grade 1, *n* = 339	1.3 (0.8 to 2.0)	0.222	1.4 (0.9 to 2.2)	0.068
Max BML grade 2, *n* = 54	3.6 (2.8 to 7.2)	**0.003**	3.9 (3.0 to 7.5)	**0.002**
Max BML grade 3, *n* = 15	3.1 (2.4 to 5.5)	**0.001**	3.2 (2.4 to 5.6)	**0.001**
Presence BML grade 0, *n* = 264	Reference		Reference	
Presence BML grade 1, *n* = 253	1.3 (0.9 to 2.1)	0.172	1.4 (0.9 to 2.1)	0.167
BML burden grade 0, *n* = 109	Reference		Reference	
BML burden grade 1, *n* = 217	1.2 (0.8 to 2.1)	0.369	1.2 (0.8 to 2.0)	0.372
BML burden grade 2, *n* = 134	1.5 (0.9 to 2.5)	0.100	1.5 (0.9 to 2.6)	0.103
BML burden grade 3, *n* = 39	5.0 (2.2 to 12.2)	**< 0.001**	5.1 (2.3 to 12.2)	**< 0.001**
BML burden grade 4, *n* = 18	4.9 (1.4 to 14.0)	**0.023**	5.0 (1.6 to 14.3)	**0.017**

BML, bone marrow lesion; CI, confidence interval

¶ Adjusted for baseline age, sex, BMI and KL grade

## Discussion

In symptomatic clinically diagnosed knees with OA, the severity of BMLs, except for those in the lateral compartment, was found to be associated with an increased risk of incident knee joint surgery over a 5-year period. Moreover, a higher risk was observed with more severe BMLs, although Max BML grade 1 seems to have no association with incident joint surgery. The association between BMLs and KL progression strengthen the significance of BMLs in the disease progression process. However, the PPVs of BMLs were found to be low, which limits their practical application in identifying cases that require joint surgery within 5 years.

The association between BMLs and incident joint surgery could be explained by the fact that BMLs causes pain and may cause OA structural progression. Patients with knee OA typically seek surgical treatment only when the joint becomes severely uncomfortable and significantly affects their daily lives [12, 13]. Joint pain is the most common symptom associated with OA. Previous studies have shown that the presence and size of OA-related BMLs are associated with joint pain [14, 15]. Specifically, the likelihood of knee pain is 2 to 5 times higher in patients with BMLs detected in their knees compared to those without BMLs [14]. The mechanisms underlying this association may involve cartilage degeneration, altered joint stress, and synovial inflammation [3]. Some studies also suggest that BMLs themselves can directly cause pain [16]. The main mechanism involves the release of nerve sensitizing factors (including cytokines and nerve growth factors) by osteoclasts and inflammatory cells within BMLs [16]. These nerve sensitizing factors stimulate peripheral pain receptors, leading to the transmission of pain signals and subsequent joint pain perception in patients [3]. On the other hand, OA is a disease that affects the entire joint, and BMLs have been found to be associated with structural progression of osteoarthritis. Roemer et al. found a strong correlation between BMLs and structural progression within the same compartment where they were located. Besides, 81% of patients with medial joint compartment progression had accompanying BMLs [17]. Our study supports their findings and additionally reveals a stronger correlation between BMLs in the patellofemoral compartment and incident surgery compared to the medial joint space. However, further research is needed to confirm the association of BMLs in the patellofemoral compartment with structural progression. It is important to note that not all patients in our study had perioperative MRI imaging results, which limited our ability to conduct correlation analyses between BMLs and structural progression.

The strong correlation we observed between BMLs and the 5-year surgery rate suggests that BMLs play a significant role in OA progression and may serve as a potential target for treatment. For instance, some drugs that improve BMLs (such as bisphosphonates [18, 19] and strontium ranelate) [20] and target BML related nerve sensitizing factors have shown some analgesic effects. However, there are currently no clinical trials investigating whether these drugs can reduce long-term OA-related surgery rates. The findings of our study support the need for such clinical trials and suggest that the optimal target population should consist of patients with BMLs in the patellofemoral compartment, while it is not recommended to include patients with mild BMLs (i.e., Max BML grade 1). Additionally, the results of our study also support the improvement of BMLs (particularly in patellofemoral compartment) as an early outcome measure for 5-year surgery rates. However, our findings do not support excessive interventions for patients who are found to have BMLs in their first MRI examinations, since the positive predictive values for all BML grades were relatively low, indicating that the majority of patients with BMLs will not progress to the stage requiring surgery within 5 years.

Several previous studies have investigated the association between BMLs and the risk of joint surgery, but these studies had methodological limitations when compared to the current study. For instance, one study examined 65 highly selected subjects with knee OA and found an increased risk of knee replacements in individuals with BMLs at the time of their initial MRI examination [21]. However, this study did not account for potential confounders and did not analyze the association separately for each joint compartment, which is important considering the compartment-specific associations observed in our study. Another study included a small group of symptomatic knee OA patients (*n* = 109) and reported a positive association between the severity of BMLs and the risk of knee joint replacement over a 4-year period, adjusting for potential confounders [9]. However, the number of joint replacement surgeries (*n* = 16) was too small to build robust statistical models and obtain precise estimates. The same research team conducted a similar study using the same study population and found an association between subchondral bone cysts and the risk of joint replacement surgery within 4 years [22]. Notably, none of these studies examined BMLs specifically in the patellofemoral compartment, while our study revealed that the maximum BML grades in the patellofemoral compartment had the highest odds ratios for incident joint surgery.

This study has several limitations that should be acknowledged. Firstly, there may be potential indication bias since patients who did not undergo MRI examinations were excluded from the study. It is possible that patients who received MRI examinations were more concerned about their health, and clinicians may have paid more attention to those knees. However, it is important to note that most clinically diagnosed OA knees in our hospital typically undergo at least one MRI examination, and the number of exclusions for this reason was small (*n* = 89) in this study. Secondly, the results and ORs reported in this study are specific to BMLs identified in the initial MRI films of patients and may not be generalizable to cases where BMLs are detected in subsequent examinations. Thirdly, due to the retrospective design of the study, we lack longitudinal data on factors such as patient weight, medication use, symptom progression, and joint structural changes, which could potentially confound our findings. Lastly, although the study included a large group of patients, the overall surgical rate over the 5-year period was low, which may have influenced the predictive values. Specifically, in populations where the disease prevalence is low, even a marker with high specificity may yield a relatively low PPV. Conversely, in such populations, the NPV of a test tends to be higher. Therefore, the interpretation of PPVs and NPVs in this study should consider the low prevalence of incidence surgery. Additionally, it’s noteworthy that the incidence of surgery is often low in OA studies, especially with short follow-up times and the inclusion of patients in early stages [14]. Future studies with a larger number of surgical cases and a longer follow-up period could help address this issue and provide more comprehensive insights.

In conclusion, among symptomatic clinically diagnosed OA knees, BMLs found in the first MRI examinations were associated with 5-year incident joint surgery, except for those allocated in lateral compartments. BMLs exhibited low PPVs but high NPVs, suggesting that individuals lacking BMLs have a low risk of undergoing joint surgery within 5 years. It’s important to note that these predictive values were determined within a context of low prevalence (7%) of incident surgery. The findings of this study call for attention to be given to the BMLs within the patellofemoral compartment as they showed the strongest association with incident surgery and exhibited the best predictive ability.

### Electronic supplementary material

Below is the link to the electronic supplementary material.

Supplementary Material 1

## Data Availability

No datasets were generated or analysed during the current study.
