# Supplementary material for: Bone marrow lesion and 5-year incident joint surgery in patients with knee osteoarthritis: a retrospective cohort study
Source: J Orthop Surg Res. 2024 May 21;19:305. doi: 10.1186/s13018-024-04705-z (PMC11107017; doi:10.1186/s13018-024-04705-z)
Supplement: Supplementary file 1 — Supplementary Material 1 [file 13018_2024_4705_MOESM1_ESM.docx]

**Appendix Table.** Baseline demographic and radiographic characteristics of the knees with/without radiographs available at 4 to 6 years (8 knees with baseline KL grade 4 were excluded).

| **Characteristics** | Radiographs available  (n=517) | Radiographs unavailable (n=486) |
| --- | --- | --- |
| Age, years, mean (SD) | 61.1 (8.8) | 61.4 (8.5) |
| Sex, female (%) | 377 (73) | 364 (75) |
| BMI, kg/m^2^, mean (SD) | 25.2 (3.7) | 25.1 (3.4) |
| Bilateral OA, yes (%) | 422 (82) | 404 (83) |
| Education, high school or higher (%) | 193 (37) | 206 (42) |
| Smoking, n (%) | 107 (21) | 69 (14) |
| Have hand OA, n (%) | 33 (6) | 35 (7) |
| Have hip OA, n (%) | 54 (10) | 47 (9) |
| Time from OA diagnosis to enrollment, months, mean (SD) | 51.9 (26.9) | 52.1 (28.2) |
| NRS pain score, 0-10, mean (SD)※ | 1.7 (1.2) | 1.8 (1.3) |
| KL grade, n (%) |  |  |
| 2 | 355 (68) | 356 (73) |
| 3 | 162 (32) | 130 (27) |

SD, standard deviation; BMI, body mass index; OA, osteoarthritis; NRS, numeric rating scale; KL, Kellgren & Lawrence.

※78 knees had missing values in NRS score.
